# Obesity and Iron Metabolism: Mechanisms, Status Assessment, and Clinical Implications

**DOI:** 10.1007/s13679-026-00753-0

**Published:** 2026-09-05

**Authors:** Sixtus Aguree, Xiangqi Meng

**Affiliations:** Department of Applied Health Science, School of Public Health- Bloomington, Indiana University, Bloomington, IN 47405 USA

**Keywords:** Obesity, Iron deficiency, Hepcidin, Serum ferritin, Iron status assessment, Inflammation

## Abstract

**Purpose of Review:**

Obesity reshapes systemic iron handling, yet the resulting iron phenotype is frequently misclassified at the bedside and in population studies. This review examines how excess adiposity disturbs iron metabolism and complicates iron-status assessment, with balanced attention to biology and measurement problems across adults and children.

**Recent Findings:**

Adipose tissue is an endocrine, inflammatory, and iron-handling organ. In obesity, interleukin-6 drives hepatic hepcidin through JAK/STAT3 signaling, while leptin, adipose hypoxia, and adipose hepcidin expression may contribute additional signals. Hepcidin degrades ferroportin, reducing duodenal iron export and limiting macrophage iron release, producing hypoferremia and iron-restricted erythropoiesis despite normal or elevated ferritin. We and others have shown that women with obesity may have higher hepcidin, ferritin, and inflammatory markers but lower serum iron. Stable-isotope and intervention studies suggest weight loss reduces inflammation and hepcidin and improves iron absorption, whereas the World Health Organization and Biomarkers of Nutrition for Development frameworks recommend interpreting ferritin relative to inflammation.

**Summary:**

Iron status in obesity spans a continuum from absolute or functional iron deficiency to sufficiency and, in some individuals, hyperferritinemia with possible dysmetabolic iron overload. Reliable assessment requires a multi-marker strategy: ferritin interpreted alongside an inflammation marker such as C-reactive protein and supported by transferrin saturation, soluble transferrin receptor, reticulocyte hemoglobin, or other context-appropriate indices. Management should address reversible inflammation through weight loss and metabolic risk reduction, use oral or intravenous iron according to the likelihood of true deficiency and hepcidin-mediated oral refractoriness, and avoid reflexive phlebotomy for dysmetabolic hyperferritinemia without confirmed overload.

**Graphical Abstract:**

**Figure:**
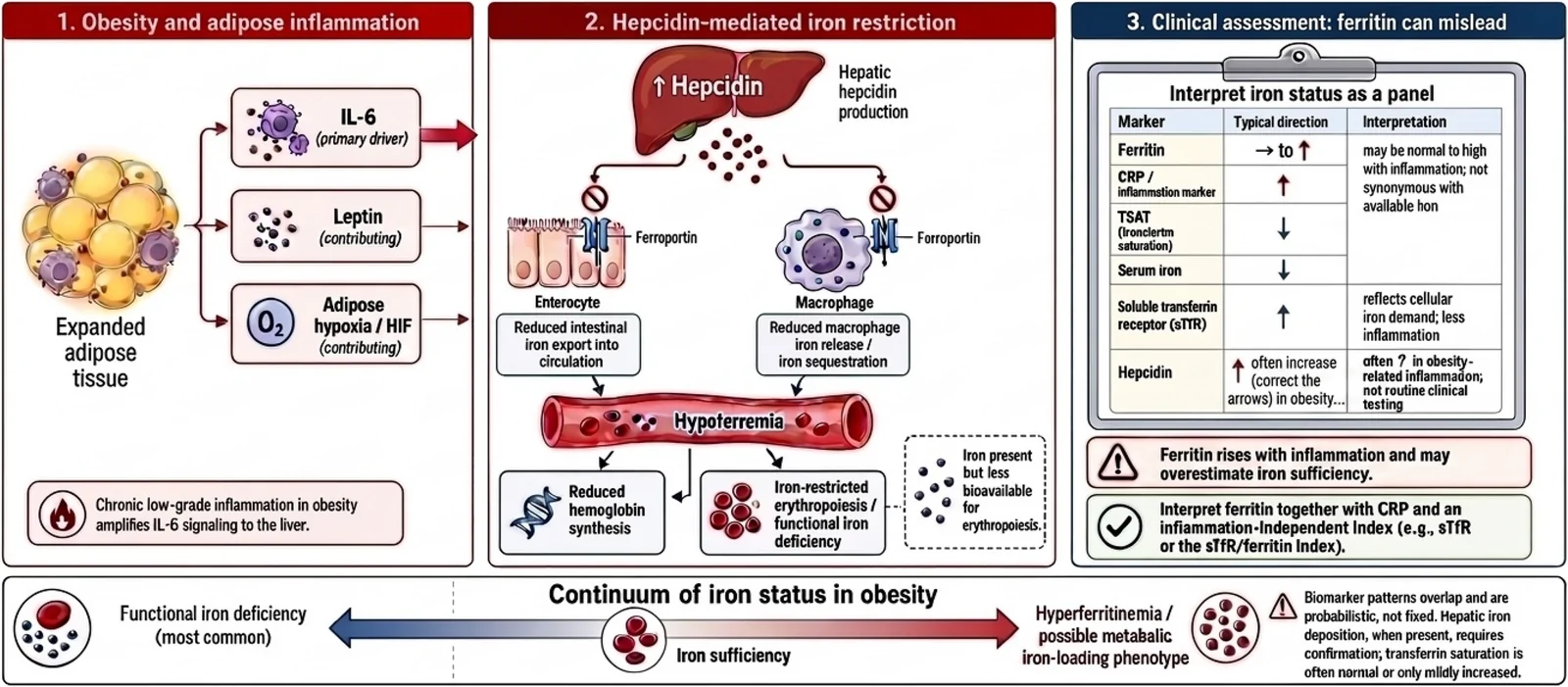
Graphical abstract of obesity-associated iron dysregulation. Expanded adipose tissue promotes chronic low-grade inflammation, with interleukin-6 as the dominant driver and leptin, adipose hypoxia, and HIF-related signaling as contributing pathways. These inflammatory signals increase hepatic hepcidin production, which inhibits ferroportin in enterocytes and macrophages. The resulting reduction in intestinal iron export and macrophage iron release causes hypoferremia, reduced hemoglobin synthesis, and iron-restricted erythropoiesis, resulting in functional iron deficiency with or without anemia. In parallel, ferritin may increase as an acute-phase reactant and may overestimate iron sufficiency when interpreted alone. Therefore, iron status in obesity should be assessed using a panel that includes ferritin, transferrin saturation, serum iron, an inflammation marker such as C-reactive protein, and an inflammation-independent index such as the soluble transferrin receptor or the soluble transferrin receptor/ferritin index. These biomarker patterns exist along a continuum from functional iron deficiency to iron sufficiency and hyperferritinemia with possible dysmetabolic iron loading. The overlap between states is common, and hepatic iron deposition requires confirmation.

## Introduction

Obesity is a major public health problem worldwide and affects approximately 40% of adults in the United States, with severe obesity more common among women and pediatric obesity remaining an important public health concern [1]. Rising obesity prevalence has direct implications for iron status because obesity-associated low-grade inflammation can impair iron homeostasis and lower circulating iron [2–4]. More than six decades ago, Wenzel and colleagues reported the unexpected finding of low serum iron in adolescents with obesity [5]; this observation later gained renewed attention as pediatric and population-based studies confirmed that adiposity is an independent risk factor for iron deficiency (ID) [6, 7]. The apparent paradox that a state of nutritional excess can predispose individuals to deficiency of an essential micronutrient has since been clarified by the discovery of hepcidin, the master iron-regulatory hormone, and by the recognition of adipose tissue as an active endocrine and immune organ rather than an inert energy depot [2, 8, 9].

The relationship is not limited to deficiency. At one end of the spectrum, obesity-related inflammation withholds iron from circulation and the developing erythron, producing functional iron deficiency (FID) and, in some patients, a blunted response to oral iron. In our study of non-pregnant women of reproductive age, women with obesity had higher hepcidin, serum ferritin (SF), and inflammatory marker levels but lower serum iron levels than normal-weight women, consistent with hepcidin-mediated iron restriction [10]. Conversely, in a subset of individuals, the same metabolic milieu is associated with hyperferritinemia and possible dysmetabolic iron loading, particularly in the setting of metabolic syndrome and metabolic dysfunction-associated steatotic liver disease (MASLD) [2, 11, 12]. Elevated ferritin in this context does not by itself establish tissue iron overload, and hepatic iron deposition requires appropriate confirmation. Iron may also feed back on adipose biology, modulating adiponectin, leptin, insulin sensitivity, and metabolic risk, although the strength of this evidence varies across experimental, observational, and clinical studies [13, 14]. Clinicians evaluating patients with obesity and abnormal iron indices therefore face a bidirectional and with a wide range of phenotypes.

A measurement problem that matters just as much in practice. Serum ferritin, the most widely available quantitative marker of body iron stores, is a positive acute-phase reactant. In chronic low-grade inflammation of obesity, ferritin can rise independently of usable iron availability, meaning that ferritin in this setting reflects inflammation in addition to body iron stores [10, 15, 16]. Therefore, a ferritin concentration within or above the reference range may obscure tissue iron deficiency or iron-restricted erythropoiesis. We have shown directly that the estimated prevalence of ID and iron-deficiency anemia (IDA) in women with obesity differs substantially depending on whether ferritin, mean cell volume (MCV), or body iron index (BII) is used to define deficiency [17]. Which iron status indices can determine whether the obesity–ID association is detected.

In this review, we provide balanced attention to the mechanisms and assessments. We first outline normal systemic iron homeostasis, then examine how obesity perturbs this system, before turning to the assessment of iron status in inflamed patients with obesity and the epidemiological, clinical, and therapeutic implications that follow.

For the review, we searched PubMed and Web of Science until June 2026 for English-language human and experimental studies on obesity and iron metabolism, hepcidin, iron-status biomarkers, and clinical assessment. We prioritized mechanistic studies, large population analyses, randomized trials, and work published in the past five years, while also including foundational earlier reports. The underlying evidence is of mixed strength, and statements throughout specify when conclusions rely primarily on mechanistic, animal, or cross-sectional data rather than controlled human trials. Key claims are supported, where possible, by multiple sources, including independent and corroborating evidence.

## Systemic Iron Homeostasis: The Hepcidin–Ferroportin Axis

### Hepcidin and Ferroportin

The systemic iron flux is predominantly governed by the hepcidin–ferroportin axis. Hepcidin, a 25-amino-acid peptide synthesized mainly by hepatocytes, was first identified as an iron- and lipopolysaccharide-inducible liver peptide and subsequently established as the central hormonal regulator of systemic iron traffic [9, 18]. Its principal iron-regulatory target is ferroportin (SLC40A1), the only known cellular iron exporter. Ferroportin is expressed on the basolateral membrane of duodenal enterocytes, iron-recycling splenic and hepatic macrophages, and iron-storing hepatocytes. Hepcidin binds to ferroportin and induces its internalization and degradation, thereby reducing the delivery of dietary, recycled, and stored iron to the plasma. When sustained, this process produces hypoferremia [9, 19].

The direction of this system is straightforward. When iron stores are low or erythropoietic demand is high, hepcidin levels fall, ferroportin is preserved, and iron flows from the diet and macrophage recycling into the transferrin pool to support erythropoiesis. Conversely, when iron stores are abundant or infection threatens, hepcidin levels increase, ferroportin is removed from the cell surface, and iron is withheld from circulation. This iron-withholding response is protective during acute infection; however, when chronically activated by inflammation rather than transient host defense, it contributes to anemia of inflammation. Obesity-related iron restriction may be viewed as a low-grade, metabolically driven variant of the inflammatory iron-withholding response [3, 8].

### Sensing Iron Stores and Inflammation

Hepcidin transcription integrates two major upstream signals: iron status and inflammation. Iron stores are primarily sensed through the bone morphogenetic protein (BMP)/SMAD pathway. BMP6 and BMP2, produced largely by liver sinusoidal endothelial cells in response to tissue iron, bind to BMP receptors and the co-receptor hemojuvelin, leading to the phosphorylation of SMAD1/5/8. These phosphorylated SMADs form complexes with SMAD4 and activate the hepcidin promoter (HAMP). HFE and transferrin receptor 2, which respond to circulating holo-transferrin, also contribute to this regulatory axis. Disruption of this pathway, including the loss of BMP6 signaling, can result in severe iron overload [20, 21].

Inflammation is primarily mediated by interleukin-6 (IL-6), which activates the JAK/STAT3 signaling pathway. Phosphorylated STAT3 binds to a responsive element in the hepcidin promoter and induces hepcidin transcription [22, 23]. These two inputs are not merely additive. The BMP/SMAD iron-sensing pathway and the IL-6/STAT3 inflammatory pathway converge at the hepcidin promoter, and their maximal induction often requires cooperation between these pathways [20, 22]. This convergence matters because chronic inflammatory signaling is layered onto the normal iron-sensing set point.

## Mechanisms Linking Obesity to Disordered Iron Metabolism

### Chronic Low-Grade Inflammation and the IL-6–Hepcidin Axis

**Fig. 1:**
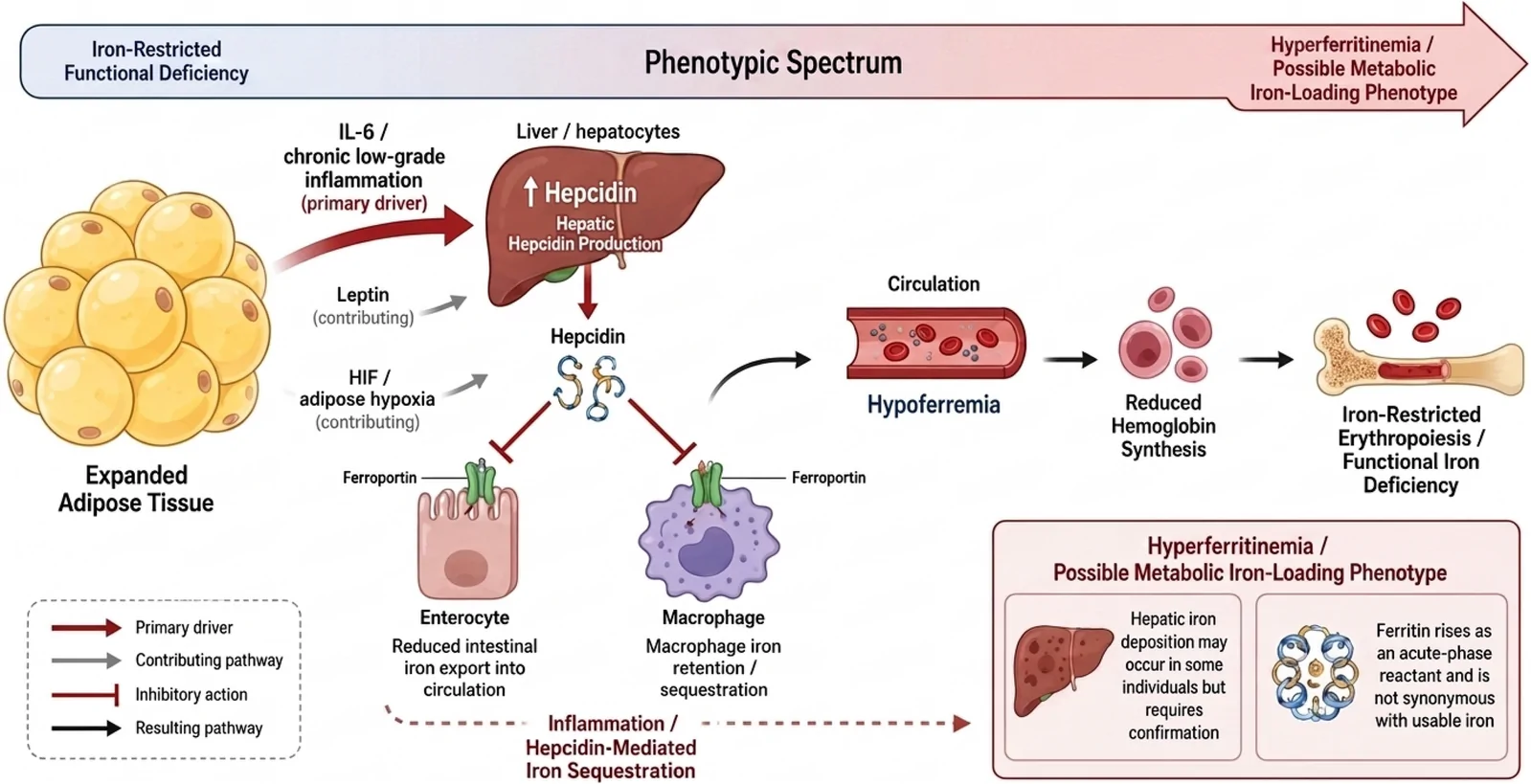
Pathophysiology of obesity-associated iron dysregulation. Expanded adipose tissue promotes chronic low-grade inflammation, with interleukin-6 as the primary driver and leptin, adipose hypoxia, and HIF-related pathways acting as contributors. These signals increase hepatic hepcidin production, leading to ferroportin inhibition and reduced iron export from duodenal enterocytes and macrophages. The resulting decrease in circulating iron produces hypoferremia, reduced hemoglobin synthesis, and iron-restricted erythropoiesis, manifesting as functional iron deficiency with or without anemia. In parallel, inflammation and hepcidin-mediated iron sequestration can raise ferritin as an acute-phase reactant, so elevated ferritin is not necessarily synonymous with usable iron. At the iron-replete end of the phenotypic spectrum, some individuals may develop hyperferritinemia with a possible metabolic iron-loading phenotype, although hepatic iron deposition requires confirmation. Based on refs [4, 8, 13], 26– [29]

A central link between obesity and iron restriction is the chronic inflammatory stimulation of hepcidin (Fig. 1). In human infusion studies, IL-6 increased urinary hepcidin more than sevenfold within hours and produced hypoferremia, establishing IL-6 as a sufficient inflammatory driver of hepcidin-mediated iron restriction [24]. Obesity provides a sustained source of this stimulus. Expanded and dysfunctional visceral adipose tissue secretes IL-6 into the portal circulation and is accompanied by elevated C-reactive protein (CRP), leptin, and other inflammatory mediators, creating a persistent signal for hepatocyte Hepcidin synthesis. Patients with obesity often show higher hepcidin concentrations that correlate positively with inflammatory markers and inversely with serum iron levels. In our cohort of women of reproductive age, women with obesity had higher hepcidin and CRP levels and lower serum iron levels than normal-weight women, consistent with hepcidin-mediated iron restriction [2, 4, 10, 25]. Because this inflammatory signal is layered onto intact BMP/SMAD iron sensing, hepcidin in obesity may be inappropriately high relative to circulating transferrin-bound iron and erythropoietic need.

### Adipose Tissue as an Iron-Handling Endocrine Organ

Adipose tissue is not merely a passive recipient of inflammatory signaling; it also actively participates in iron regulation. Bekri et al. showed that human adipose tissue expresses hepcidin mRNA and protein, that adipose hepcidin expression is increased in severe obesity, and that it correlates with IL-6 and CRP. IL-6 also induces adipose hepcidin expression in vitro, leading the authors to propose that hepcidin is a proinflammatory adipokine that may contribute to the hypoferremia of obesity [26].

Leptin, which is secreted in proportion to fat mass, may provide additional adipose-derived signals. In hepatoma cells, leptin induced hepcidin expression through the Ob-Rb receptor and the JAK2/STAT3 pathway, an effect abolished by JAK2 inhibition or disruption of the STAT3 binding site [30]. Adipose tissue may contribute to systemic iron restriction both indirectly, by promoting hepatic hepcidin production through inflammatory and adipokine signaling, and potentially directly, through local adipose hepcidin expression. IL-6 remains the better-established driver of obesity-related hepcidin induction, while leptin and adipose hepcidin are best regarded as contributory.

### Adipose Tissue Iron Handling and the Macrophage Switch

A related counterintuitive process occurs within the fat depots. Adipose tissue macrophages can function as local iron-buffering cells, taking up and recycling iron via an iron-handling phenotype. In obesity, the macrophage iron-handling program appears to shift. Experimental and translational studies suggest that adipose tissue macrophages may become relatively iron-refractory, sequester less iron, and lose expression of iron-recycling genes, allowing iron to redistribute into adipocytes [27, 31, 32]. This process can produce adipocyte iron loading, despite systemic iron restriction.

Local adipocyte iron loading may have metabolic effects. It provides one mechanism by which iron can influence adipocyte function and whole-body metabolism, as summarized in recent syntheses of adipose tissue iron biology [33]. However, the strength of evidence varies across experimental models and human studies, and the causal relevance of adipose iron redistribution in human obesity remains an active area of investigation.

### Impaired Dietary Iron Absorption

The downstream consequence of elevated hepcidin is ferroportin loss from duodenal enterocytes and reticuloendothelial macrophages. In enterocytes, hepcidin reduces basolateral iron export into the circulation, and in macrophages, it traps recycled iron within the reticuloendothelial system. The net effect is reduced plasma iron and iron-restricted erythropoiesis despite normal or increased stored iron, a pattern characteristic of functional iron deficiency [10, 34].

Stable isotope absorption studies support the clinical relevance of this mechanism. Dietary iron absorption is reduced in women who are overweight and obese, and the absorption-enhancing effect of ascorbic acid may be attenuated [35]. This mismatch reflects the different sites at which these factors act. Ascorbic acid primarily enhances luminal non-heme iron reduction and uptake at the apical surface of enterocytes, whereas hepcidin restricts iron export at the basolateral membrane through ferroportin degradation. Therefore, luminal enhancers may only partially overcome the hepcidin-mediated restriction.

Iron requirements may also be higher in some individuals with obesity because greater blood volume and hemoglobin mass can increase total body iron needs, complicating the interpretation of concentration-based iron biomarkers [36]. Diet composition may also contribute independently. High-fat feeding can impair duodenal iron uptake through hepcidin-independent reductions in mucosal transport, suggesting that impaired absorption in obesity is multifactorial [37]. In addition, unabsorbed luminal iron interacts bidirectionally with the gut microbiome, which is altered in obesity. This host–microbe interface is an emerging and incompletely understood modifier of iron metabolism in this population [38].

### Iron, Adipokines, and Metabolic Dysfunction

The relationship between obesity and iron is bidirectional because iron in metabolic tissues can shape adipokine signaling and insulin sensitivity. Gabrielsen et al. showed that iron loading in adipocytes suppresses adiponectin transcription through a FOXO1-dependent mechanism. In mice, adipocyte-specific loss of ferroportin results in adipocyte iron loading, reduced adiponectin levels, and insulin resistance. In humans, serum ferritin levels are inversely correlated with adiponectin levels, independent of inflammation [13]. Iron may also repress leptin transcription through cyclic-AMP response element-binding protein, so that higher adipocyte iron lowers leptin and, in animal models, increases food intake independently of body weight [14].

Excess iron also promotes the generation of reactive oxygen species and lipid peroxidation. Using β2-microglobulin knockout mice with iron overload and high-fat feeding, we found that dietary phytic acid attenuated oxidative stress, supporting the concept that the metabolic cost of tissue iron is mediated in part through redox injury and may be modifiable [39]. Hepatic iron handling is also influenced by insulin signaling, providing a potential route by which insulin resistance contributes to the iron accumulation observed in fatty liver [2, 12]. Together, these findings suggest that iron is not only a downstream consequence of obesity-related inflammation but may also contribute actively to adipose dysfunction, oxidative stress, and insulin resistance. However, the extent to which these mechanisms drive human metabolic diseases requires further clarification.

### Dysmetabolic Hyperferritinemia and Dysmetabolic Iron Overload Syndrome

At the iron-replete end of the obesity-related iron spectrum lies dysmetabolic hyperferritinemia and, in some individuals, dysmetabolic iron overload syndrome (DIOS). DIOS is generally characterized by hyperferritinemia, normal or modestly increased transferrin saturation, features of metabolic syndrome or metabolic dysfunction-associated steatotic liver disease (MASLD), and evidence of mild hepatic iron accumulation after exclusion of classical causes such as hereditary hemochromatosis [11, 12].

The reported prevalence varies substantially according to the diagnostic criteria, population, and whether hepatic iron is directly measured. Therefore, hyperferritinemia in obesity or MASLD should not be assumed to represent true iron overload without appropriate confirmation. In many studies, hepcidin levels are normal or elevated in DIOS, suggesting that iron loading may reflect impaired hepcidin activity, relative ferroportin resistance, altered macrophage iron handling, or metabolic liver injury rather than simple hepcidin deficiency. Other studies suggest that hepcidin may be inadequate relative to the degree of iron loading, but this question remains unresolved [11, 12]. In fatty liver, hepatic iron content and serum hepcidin are among the determinants of serum ferritin, which has been associated with insulin resistance and histological liver injury [40]. Thus, dysmetabolic hyperferritinemia and DIOS should be viewed as possible iron–replete or iron-loaded phenotypes within the broader obesity-related iron–inflammation spectrum. However, their mechanisms are not identical to those of functional iron deficiency, and ferritin alone cannot distinguish between inflammation, metabolic liver injury, and true tissue iron overload.

### Adipose Hypoxia, the HIF Pathway, and Iron

A further mechanism linking iron, adipose biology, and insulin resistance involves the hypoxia-inducible factor (HIF) pathway, the stability of which is iron-dependent. Under normoxia, HIF transcription factors are marked for degradation by prolyl hydroxylase domain enzymes, which require molecular oxygen and ferrous iron as cofactors [28, 41]. Thus, HIF signaling is jointly regulated by oxygen availability and iron-dependent enzymatic activity. In murine models, intestinal HIF-2α is an important transcriptional regulator of duodenal iron absorption, driving the expression of divalent metal transporter 1, duodenal cytochrome b, and ferroportin. It is also required for the normal absorptive response to iron deficiency. Hepcidin may restrain absorption, in part, by suppressing the enterocyte HIF-2α axis [28, 41]. These data come largely from genetic mouse models, and a direct role of intestinal HIF-2α in human obesity-related iron restriction has not been established. Therefore, this coupling between hepcidin signaling and intestinal absorptive pathways should be considered mechanistically plausible rather than proven in human obesity.

In adipose tissue, rapid expansion of fat depots can outstrip their vascular supply and produce local hypoxia. Stabilization of HIF-1α has been associated with adipose fibrosis and inflammation, partly through lysyl oxidase-mediated collagen cross-linking, while disruption of adipose HIF-1α improves insulin signaling and reduces fat mass in high-fat-fed mice [29]. Because prolyl hydroxylase activity depends on iron, perturbations of adipocyte iron, whether from adipocyte iron loading or iron-driven oxidative stress, could plausibly alter HIF tone. Therefore, adipose hypoxia may be the point at which disordered iron handling, inflammation, and insulin resistance converge. However, this hypothesis has not yet been directly tested in humans [13, 29, 31].

## The Obesity–Iron Paradox and Assessing Iron Status

### The Obesity–Iron Paradox and its Clinical Spectrum

The phenotype that emerges from obesity-related iron dysregulation is only superficially contradictory to the above-mentioned mechanisms. A common pattern in patients with obesity is normal or elevated serum ferritin (SF) levels together with low serum iron and low transferrin saturation (TSAT) levels, which indicates apparent iron sufficiency by one marker but restricted circulating iron by another. This is because ferritin in this setting reflects inflammation as well as storage iron, whereas low TSAT reflects reduced circulating iron availability, often mediated by hepcidin-driven sequestration of iron away from plasma and the developing erythron [2, 10, 15].

In our cross-sectional cohort of young women of reproductive age (*n* = 47), serum iron correlated negatively with ferritin levels among women with obesity (*r* = − 0.46, *p* = 0.030) but positively among women with normal weight (*r* = 0.65, *p* = 0.002) [10]. This finding illustrates that a normal or high ferritin concentration can coexist with restricted iron availability when inflammation is present, although the small sample size and cross-sectional design warrant cautious interpretation.

**Fig. 2:**
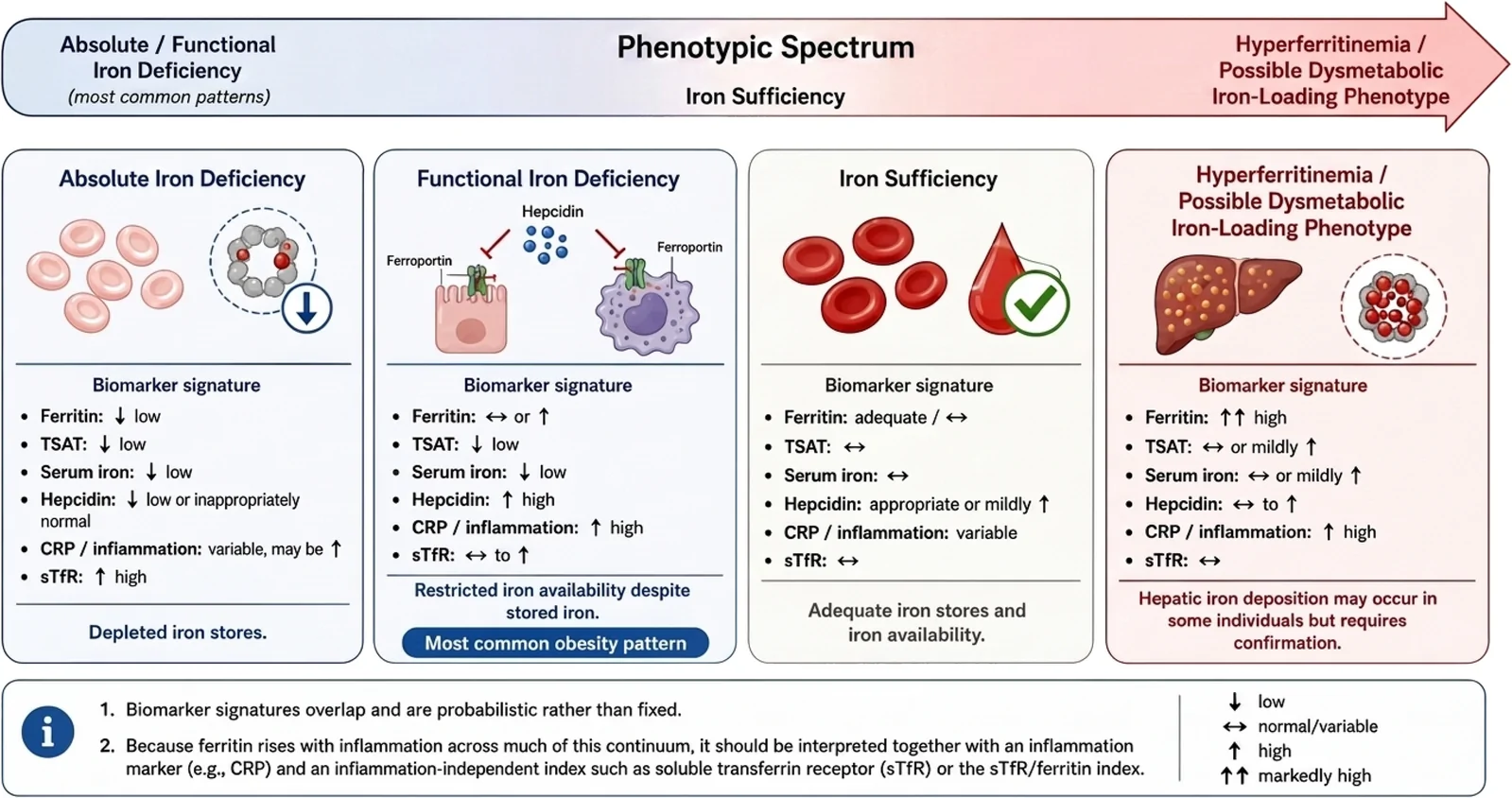
The continuum of iron status in obesity. Obesity-associated iron dysregulation spans a phenotypic continuum from absolute and functional iron deficiency, which represents the most common patterns, through iron sufficiency to hyperferritinemia with possible dysmetabolic iron loading. Each state is characterized by a typical biomarker signature, although these patterns overlap and should be interpreted as probabilistic rather than fixed. Because serum ferritin may rise with inflammation across much of this continuum, it should be interpreted together with an inflammation marker, such as C-reactive protein, and an inflammation-independent index, such as soluble transferrin receptor or the soluble transferrin receptor/ferritin index. Based on refs [10– [12, 14, 16, 34]

Several iron phenotypes that need to be told apart (Table 1). Absolute iron deficiency reflects genuinely depleted stores and is typically characterized by low SF, low TSAT, increased total iron-binding capacity (TIBC), and elevated soluble transferrin receptor (sTfR). Functional iron deficiency, a common obesity-associated pattern, reflects restricted iron availability despite preserved or increased storage iron, resulting in normal-to-high SF, low TSAT, and elevated or inappropriately high hepcidin levels. Anemia of inflammation represents a more severe or clinically apparent expression of the same iron-withholding biology, usually with low serum iron and TSAT levels, normal or elevated ferritin levels, and high hepcidin levels [3, 34]. Because absolute and functional iron deficiency may coexist in the same patient (Fig. 2), single-marker interpretation is often inadequate. Careful assessment instead draws on several biomarkers that capture storage iron, circulating iron supply, erythroid iron demand, inflammation, and erythropoietic iron availability in the body.

**Table 1:** Biomarker patterns distinguishing the principal iron states encountered in obesity [10, 11, 14, 16, 34]

Iron state	SF	TSAT	sTfR	Hepcidin	Typical obesity context
Absolute iron deficiency	Low	Low	High	Low or inappropriately normal	Blood loss, low intake, menstruation, pregnancy, post-bariatric malabsorption
Functional iron deficiency / iron-restricted erythropoiesis	Normal or high	Low	Normal, variable, or mildly high	High	Common obesity-associated pattern driven by inflammation and hepcidin-mediated iron sequestration
Anemia of inflammation	Normal or high	Low	Usually normal	High	More severe or chronic inflammatory burden; may coexist with absolute iron deficiency
Dysmetabolic hyperferritinemia / possible DIOS	High	Usually normal; sometimes mildly high	Usually normal	Normal or high	Metabolic syndrome and MASLD; hepatic iron loading may occur but requires confirmation
Iron sufficiency	Normal	Normal	Normal	Appropriate for iron status	Healthy iron balance without biochemical evidence of deficiency or overload

*SF* serum ferritin, *TSAT* transferrin saturation, *sTfR* soluble transferrin receptor, *DIOS* dysmetabolic iron overload syndrome, *MASLD* metabolic dysfunction-associated fatty liver disease. Patterns overlap and should be interpreted as probabilistic rather than deterministic. In particular, sTfR is typically normal in pure anemia of inflammation but may increase when absolute iron deficiency coexists or when the erythroid iron demand is high

### The Biomarker Toolbox and What Each Marker Reflects

**Fig. 3:**
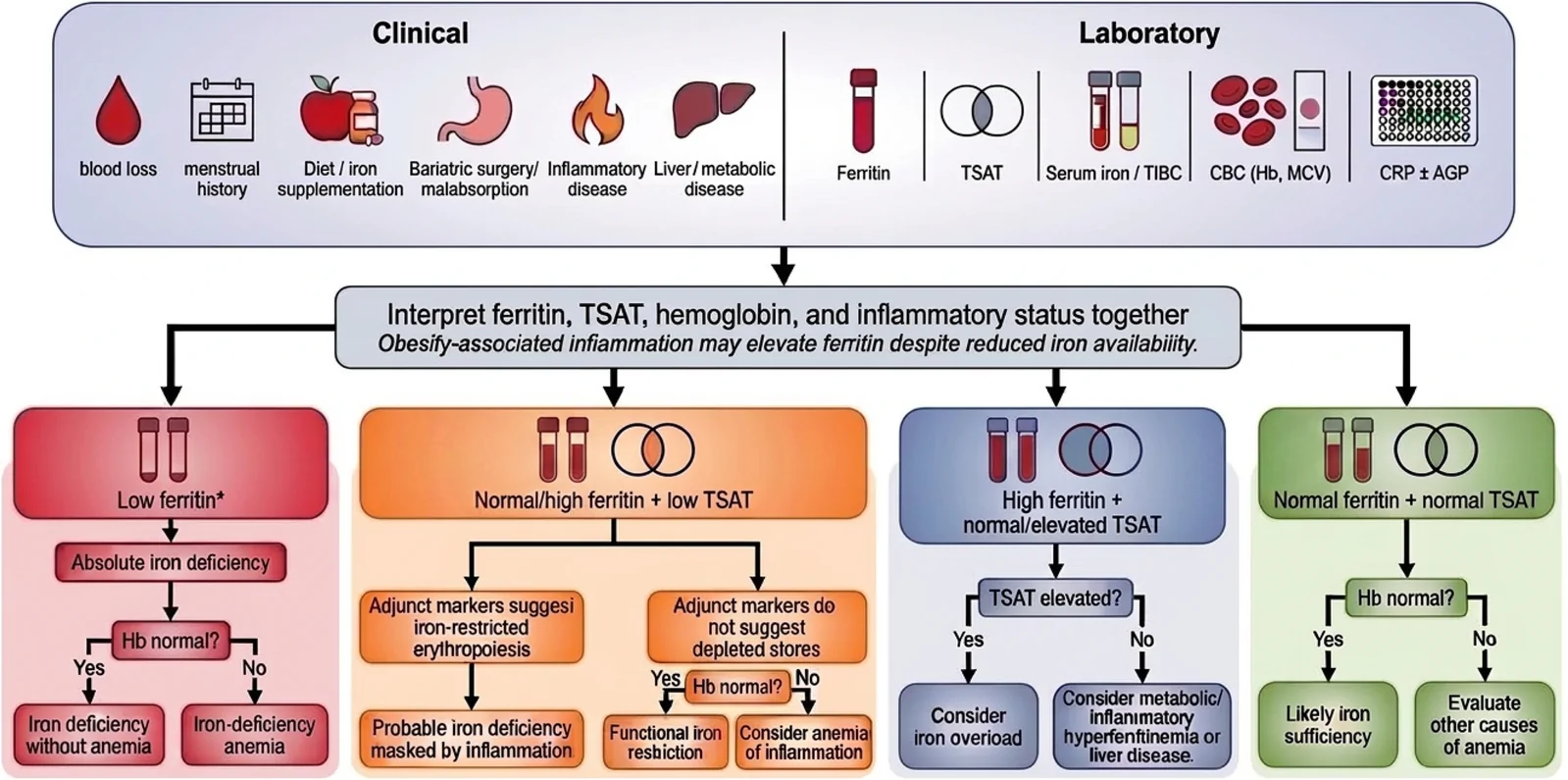
Iron-status assessment in patients with obesity. This framework summarizes iron-status interpretation in obesity using ferritin, transferrin saturation (TSAT), hemoglobin, and inflammatory status. Because obesity-associated inflammation may elevate ferritin independently of iron stores, ferritin should not be interpreted alone. Low ferritin supports absolute iron deficiency, whereas normal/high ferritin with low TSAT may indicate iron-restricted erythropoiesis or functional iron restriction. When results are inconclusive, sTfR, Ret-He/CHr, or the sTfR/log ferritin index may provide additional information. Elevated ferritin with elevated TSAT warrants consideration of iron overload; elevated ferritin without elevated TSAT may reflect metabolic/inflammatory hyperferritinemia or liver disease. Abbreviations: AGP, α1-acid glycoprotein; CBC, complete blood count; CRP, C-reactive protein; Hb, hemoglobin; MCV, mean corpuscular volume; Ret-He/CHr, reticulocyte hemoglobin equivalent/content; sTfR, soluble transferrin receptor; TIBC, total iron-binding capacity; TSAT, transferrin saturation

No single test captures iron status across its full biological range, and each available biomarker reports a different compartment of iron metabolism (Table 2; Fig. 3). Using two or more iron indices is recommended when assessing iron deficiency, particularly in inflammatory states [42]. Serum ferritin is the best and most widely available quantitative marker of storage iron under non-inflammatory conditions. However, because ferritin is a positive acute-phase reactant, its specificity for iron stores is reduced during inflammatory states [42]. In obesity, ferritin levels may be normal or elevated even when circulating iron availability is restricted. TSAT reflects the proportion of transferrin occupied by iron and serves as a practical indicator of the circulating iron available for tissue delivery. It falls under both absolute iron deficiency and functional iron deficiency, but it does not distinguish between depleted stores and inflammation-mediated sequestration.

TIBC, an indirect measure of transferrin concentration, is typically increased in absolute iron deficiency but may be normal or reduced in inflammation because transferrin is a negative acute phase reactant. Thus, TIBC can help differentiate absolute deficiency from inflammatory iron restriction when interpreted in conjunction with ferritin and TSAT. The sTfR reflects cellular and erythroid iron demand. It is less affected by inflammation than ferritin and is therefore useful in inflamed patients, although the cutoffs are assay-specific and sTfR may vary with erythropoietic activity [43, 44].

The body iron index, commonly calculated as sTfR/log ferritin, combines information on tissue iron demand and storage iron and may outperform either component alone in some settings. However, because the index includes ferritin, it is not entirely free from inflammatory confounders and should be interpreted in a clinical context. Reticulocyte hemoglobin content, reported as CHr or Ret-He depending on the analyzer, provides a near-real-time measure of iron available for erythropoiesis and can decrease before hemoglobin levels decline [45]. It is useful for detecting iron-restricted erythropoiesis, although the cutoffs vary by analyzer and population.

Hepcidin directly indexes the regulatory pathway responsible for inflammation-mediated iron restriction in the body. In obesity, elevated or inappropriately high hepcidin levels support the mechanism of impaired ferroportin-mediated iron export and reduced iron availability. However, hepcidin assays have not yet been standardized for routine clinical decision-making in many settings. In contrast, hemoglobin is a late and non-specific biomarker. It detects anemia but does not define iron status by itself because anemia may result from iron deficiency, inflammation, renal disease, hemoglobinopathy, nutritional deficiencies, or other causes.

**Table 2:** Iron status biomarkers in obesity: biological meaning, inflammatory confounding, expected behavior, and practical interpretation [8, 15], 43– [45]

Biomarker	What it reflects	Inflammation-affected?	Behavior in inflammation/obesity	Practical role in obesity
Serum ferritin (SF)	Iron stores under non-inflammatory conditions	Yes	Positive acute-phase reactant; may rise independently of iron stores	Interpret with CRP or another inflammation marker, and with TSAT and/or sTfR when inflammation is present
Transferrin saturation (TSAT)	Circulating iron available for tissue delivery	Yes, indirectly	Often low in inflammation and in both absolute and functional ID	TSAT < 20% supports restricted iron availability, especially when ferritin is normal or elevated
Total iron-binding capacity (TIBC) / transferrin	Transferrin concentration and iron-binding capacity	Yes	Typically high in absolute ID; normal or low in inflammation because transferrin is a negative acute-phase reactant	Helps distinguish absolute ID from inflammatory iron restriction when interpreted with ferritin and TSAT
Soluble transferrin receptor (sTfR)	Cellular and erythroid iron demand	Minimally / less affected	Usually increases in absolute ID; often normal in anemia of inflammation, but may rise when true ID coexists	Useful inflammation-resistant marker of tissue iron need; cutoffs are assay-specific
sTfR/log ferritin index	Cellular iron demand relative to iron stores	Less affected than ferritin alone, but not fully inflammation-independent	Often improves detection of absolute ID when inflammation coexists	Helps distinguish absolute ID, functional ID, and mixed patterns; interpretation is assay- and cutoff-dependent
Reticulocyte hemoglobin content (CHr/Ret-He)	Recent iron availability for erythropoiesis	Minimal	Falls early when iron delivery to marrow is restricted	Useful early marker of iron-restricted erythropoiesis; approximate cutoffs around 25–30 pg, often 28–29 pg, are analyzer- and population-dependent
Hepcidin	Central regulatory signal restricting iron export through ferroportin	Yes	Often elevated in obesity-related inflammation through IL-6–mediated signaling; leptin and adipose hypoxia may contribute	Mechanistic marker and potential predictor of poor oral-iron response; not yet standardized for routine clinical decision-making
Hemoglobin (Hb)	Presence and severity of anemia	Indirect	Falls late and is non-specific	Detects anemia but does not define iron status by itself

*CRP* C-reactive protein, *Hb* hemoglobin, *ID* iron deficiency, *Ret-He* reticulocyte hemoglobin equivalent. *SF* serum ferritin, *sTfR* soluble transferrin receptor, *TSAT* transferrin saturation

### The Core Problem: Ferritin as an Acute-Phase Reactant

The central pitfall in assessing iron status in obesity is over-reliance on ferritin. Proinflammatory cytokines associated with adipose tissue dysfunction can induce hepatic and macrophage ferritin synthesis; consequently, ferritin may increase with adiposity and inflammation even when circulating iron availability decreases [16]. In a representative cross-sectional study, mean ferritin increased stepwise across normal-weight, overweight, and obesity categories, whereas transferrin saturation (TSAT), serum iron, and hemoglobin decreased and C-reactive protein (CRP) increased [15]. These findings support the view that, in obesity, ferritin may reflect inflammatory burden as well as iron stores and therefore cannot be interpreted as a stand-alone marker of iron sufficiency.

The practical magnitude of this distortion may be substantial. In the pooled Biomarkers Reflecting Inflammation and Nutritional Determinants of Anemia (BRINDA) analysis of more than 50,000 individuals, adjusting for inflammation increased the estimated prevalence of depleted iron stores. The median absolute increase ranged from approximately 7–25% points in preschool children and 2–8% points in women of reproductive age, with larger shifts in settings with a greater inflammatory burden [46]. Thus, a ferritin concentration within the conventional reference range should not automatically be interpreted as an adequate iron status in patients with obesity and elevated inflammatory markers.

Conversely, even within its broad reference interval, ferritin may behave less as a simple deficiency/sufficiency threshold and more as a continuous marker influenced by iron stores, inflammation, insulin resistance, liver injury, and metabolic risk. Values toward the upper end of the normal range or elevated values may indicate inflammation and metabolic dysfunction rather than protective iron sufficiency alone [15, 40]. A single ferritin cutoff cannot capture this behavior.

### Why the Choice of Iron Model Matters

Because ferritin behaves as an acute-phase reactant, the estimated burden of iron deficiency in obesity strongly depends on the combination of markers used. In NHANES 2001–2006, we estimated iron deficiency among non-pregnant women aged 20–49 years using three standard population models. The prevalence of iron deficiency in women with obesity versus normal weight was 22.9% versus 12.5% by the ferritin model, 20.0% versus 9.0% by the mean cell volume (MCV) model, and 10.5% versus 8.1% by the body iron index (BII) model. The prevalence of anemia was 9.3% versus 5.5% [17].

These findings illustrate that ferritin- and MCV-based definitions may yield a larger apparent obesity–iron deficiency gap, whereas the BII model may narrow this gap. Therefore, the same underlying data can support different conclusions depending on the iron status model selected. For population studies, this is a central methodological message: the choice of iron indices strongly influences the estimated burden of iron deficiency in obesity and should be specified, justified, and harmonized before estimates are compared across studies or pooled in meta-analyses [17, 47].

### Correcting Ferritin for Inflammation

Consensus guidance now directly addresses this issue. The 2020 World Health Organization guidelines recommend that ferritin levels should not be interpreted using standard cutoffs alone in individuals with evidence of inflammation. Instead, investigators may raise the ferritin threshold used to define deficiency or apply mathematical correction approaches, particularly when CRP is greater than 5 mg/L or α1-acid glycoprotein is greater than 1 g/L [48]. The Biomarkers of Nutrition for Development (BOND) iron review similarly identified inflammation as a principal confounder of ferritin interpretation and provided a framework for selecting and combining iron biomarkers [42].

Two quantitative correction approaches are commonly used in population studies. The Thurnham approach applies fixed multiplicative correction factors according to the phase of the inflammatory response, as defined by CRP and α1-acid glycoprotein [49]. The BRINDA approach models the relationship between ferritin and continuous concentrations of acute-phase proteins and removes the estimated inflammatory contribution [46, 50]. The BRINDA method has the advantage of capturing graded inflammation rather than imposing discrete inflammatory categories, although both methods depend on assumptions about the relationship between inflammation and ferritin and are primarily designed for population-level estimation rather than individual clinical diagnosis.

In clinical practice, this principle is often operationalized using higher ferritin thresholds when inflammation is present. For example, ferritin below 30 µg/L is commonly used to improve sensitivity for iron deficiency in adults, whereas in overt inflammatory disease, ferritin below 100 µg/L with TSAT below 20% is often used to support iron deficiency or iron-restricted erythropoiesis [51, 52]. These thresholds are useful but context-dependent, and should not be applied mechanically across all patients with obesity without considering CRP, TSAT, sTfR, reticulocyte hemoglobin, comorbid inflammatory disease, liver disease, renal disease, and the clinical picture.

### Inflammation-Resistant Markers and a Practical Framework

Because ferritin levels are difficult to interpret in obesity, they should be measured alongside markers that capture inflammation and iron availability. Soluble transferrin receptor (sTfR) is a particularly useful complementary marker because it rises with cellular and erythroid iron demand and is less affected by inflammation than ferritin [43, 44]. Thus, an elevated sTfR in a patient with obesity and a normal or elevated ferritin supports true tissue iron deficiency or mixed absolute and functional deficiency. However, sTfR is not without limitations: it may increase with expanded or stimulated erythropoiesis, hemolysis, or other conditions that increase marrow activity, and sTfR assays lack a universal reference standard. Therefore, the reported cutoffs are method-specific and not interchangeable across laboratories.

The body iron index, calculated from sTfR and log ferritin, integrates erythroid iron demand with storage iron and may outperform either component alone in some settings. However, because the index includes ferritin, it is not entirely independent of inflammatory confounding and should be interpreted alongside CRP or other inflammation markers. Reticulocyte hemoglobin content, reported as CHr or Ret-He depending on the analyzer, provides a near-real-time automated readout of the iron available for erythropoiesis. It is relatively insensitive to inflammation and can identify iron-restricted erythropoiesis before hemoglobin levels fall, although commonly used thresholds near 28–29 pg are analyzer- and population-dependent and should not be transferred uncritically between platforms [45].

Another often-overlooked issue is that concentration-based biomarkers may be influenced by the plasma volume. Physiologic hemodilution across the menstrual cycle, especially during pregnancy, can lower measured iron concentrations independently of total body iron. These dynamics may also be altered in obesity, where greater blood volume and hemoglobin mass can shift measured concentrations and total iron requirements [36, 53, 54]. Direct hepcidin measurement is attractive in principle because it indexes the regulatory mechanism that restricts iron export and may help predict poor response to oral iron, but lack of assay standardization and limited clinical availability currently constrain its routine use [8, 16]. Bone marrow iron remains the diagnostic gold standard but is invasive and is reserved for unresolved or complex cases [34]. In practice, ferritin levels should not be interpreted alone in patients with obesity. It should be paired with an inflammation marker, such as CRP with or without α1-acid glycoprotein, and a marker of circulating or tissue iron availability, such as TSAT, sTfR, BII, or reticulocyte hemoglobin. Normal ferritin levels in the presence of elevated CRP, low TSAT, and either elevated sTfR or low reticulocyte hemoglobin should raise concern for iron-restricted erythropoiesis or mixed absolute and functional iron deficiency rather than being read as simple iron sufficiency [16, 50, 51].

This framework is intended to aid in the interpretation rather than function as a fully validated diagnostic algorithm for obesity. Many specific cutoffs have been extrapolated from populations with heart failure, inflammatory bowel disease, chronic kidney disease, or other inflammatory conditions, but they have not been prospectively validated across obesity phenotypes. Therefore, biomarker patterns should be interpreted with clinical judgment, attention to comorbidities, and, when appropriate, confirmation by response to therapy or additional testing. With this caveat, multi-marker interpretation is the key practical point for assessing iron status in obesity.

## Epidemiology and Clinical Consequences

### Prevalence of Iron Deficiency Across Body Mass Categories

The epidemiological association between adiposity and iron deficiency (ID) is robust, although its magnitude varies according to the population, age, sex, inflammation burden, and biomarkers used to define deficiency. Building on Wenzel’s original observation [5], Pinhas-Hamiel and colleagues reported low serum iron in 38.8% of children and adolescents with obesity, 12.1% of those with overweight, and 4.4% of those with normal weight, with serum iron correlating inversely with body mass index (BMI) [6]. Using United States national survey data, Nead and colleagues showed that ID increased stepwise across weight categories, from 2.1% in children with normal weight to 5.3% in those at risk for overweight and 5.5% in those with overweight; children with overweight had approximately twice the adjusted odds of ID compared with children with normal weight [7].

The key mechanistic evidence came from Cepeda-Lopez et al., who showed that higher rates of ID in Mexican women and children with obesity were predicted by obesity-related inflammation rather than by lower dietary iron intake [55]. A quantitative meta-analysis confirmed the association between overweight/obesity and ID, reporting a pooled odds ratio of 1.31 (95% CI, 1.01–1.68). This association was attenuated when ID was defined using ferritin, consistent with the diagnostic masking expected when ferritin levels rise as an acute-phase reactant [47]. In that meta-analysis, obesity was also accompanied by lower serum iron (weighted mean difference − 8.37 µg/dL) and reduced transferrin saturation (− 2.34%), indicating diminished circulating iron despite preserved or elevated stores. Our NHANES analysis extended this work by showing that the magnitude of the obesity–ID gap in women of reproductive age depends on the iron status model used [17]. Cross-population work also indicated that higher adiposity is associated with reduced iron absorption and a diminished response to iron fortification [56].

### Consequences of Iron Deficiency in Obesity

Iron deficiency in obesity has clinically significant consequences. Beyond iron-restricted erythropoiesis and overt iron deficiency anemia (IDA), ID may impair attention, memory, executive function, physical work capacity, and aerobic performance, and it may contribute to fatigue even before anemia is present [57]. Common clinical manifestations include reduced exercise tolerance, exertional dyspnea, tachycardia, and fatigue, although these symptoms are non-specific and may also reflect cardiopulmonary disease, deconditioning, sleep-disordered breathing, thyroid disease, or other comorbidities common in obesity [17, 57].

Iron is also a cofactor for thyroid peroxidase; therefore, deficiency may worsen thyroid-related symptoms or interact with hypothyroidism in susceptible individuals. Central nervous system iron deficiency is implicated in restless legs syndrome, providing another clinically relevant pathway by which ID can affect quality of life [57]. ID disproportionately affects preschool children and women of reproductive age, groups in whom obesity and severe obesity also carry major public health implications [10]. Because functional deficits can arise before hemoglobin levels fall, accurate detection of ID and iron-restricted erythropoiesis in patients with obesity is preferable to waiting for overt anemia.

### Consequences of Iron Excess and Metabolic Risk

At the iron-replete end of the obesity-related iron spectrum, hyperferritinemia and dysmetabolic iron loading are associated with cardiometabolic and hepatic risks. Dysmetabolic iron overload syndrome (DIOS), when confirmed, is linked to metabolic syndrome and MASLD, and hepatic iron deposition has been associated with more severe liver injury in some studies [12, 40]. However, elevated ferritin levels in obesity or MASLD do not prove tissue iron overload, because ferritin may reflect inflammation, metabolic dysfunction, hepatocellular injury, or a combination of these processes.

Body iron stores have also been associated with incident type 2 diabetes. In meta-analytic data, individuals in the highest ferritin category had a higher risk of type 2 diabetes than those in the lowest category, and higher heme iron intake was also associated with an increased risk [2, 58]. These observations suggest that iron-related biology may contribute to metabolic risk, although residual confounding by diet, adiposity, inflammation, liver disease, and other factors cannot be excluded in observational studies. Elevated ferritin levels are also associated with metabolic syndrome and several of its components [11].

These findings place the upper end of the obesity–iron spectrum within a state of metabolic hyperferritinemia and, in a subset of patients, possible or confirmed dysmetabolic iron loading with clinical significance. Therefore, hyperferritinemia in an obese patient should prompt an assessment of transferrin saturation, inflammatory markers, liver enzymes, MASLD/metabolic risk, and, when indicated, evaluation for hereditary hemochromatosis or hepatic iron deposition. It should not lead to reflexive reassurance or venesection without confirming the underlying cause.

## Special Populations

### Pregnancy Complicated by Obesity

Pregnancy magnifies the obesity–iron interaction in pregnant women. Under normal conditions, maternal hepcidin levels fall during gestation, increasing iron bioavailability for maternal erythropoiesis and placental transfer. In pregnancies complicated by obesity, low-grade inflammation may blunt this adaptation by maintaining relatively higher maternal hepcidin levels, thereby reducing dietary iron absorption and potentially limiting placental iron transfer. Maternal obesity has also been associated with altered maternal iron status and changes in placental iron-handling pathways, including transferrin receptor, ferroportin, and hepcidin-related signaling, although findings vary across studies and may depend on gestational age, maternal iron status, inflammation, and fetal demand [59]. Newborns of mothers with obesity may have lower iron endowment, as reflected in lower cord blood iron indices, altered cord blood hepcidin and erythropoietin levels, or other markers of impaired fetal iron status [60]. These observations support the concept that maternal obesity and excessive gestational weight gain can affect fetal iron transfer, but the mechanisms are complex and not fully explained by maternal ferritin alone [60].

Assessment during pregnancy is further complicated by physiological plasma volume expansion, which dilutes concentration-based biomarkers. Our meta-analysis quantified the magnitude and trajectory of this expansion across healthy pregnancies, and standard ferritin thresholds in pregnancy remain debated [53, 61]. Pregnancy also substantially increases iron requirements to support maternal erythrocyte expansion and fetal-placental growth. Therefore, the inflammation-related elevation of ferritin levels in maternal obesity may mask the true iron deficiency or iron-restricted erythropoiesis. In practice, iron assessment in pregnant patients with obesity should combine ferritin with transferrin saturation and an inflammation marker, and, where available, an inflammation-resistant index, such as soluble transferrin receptor or reticulocyte hemoglobin. As fetal iron is essential for neurodevelopment, careful assessment and treatment of maternal iron deficiency in this group may have maternal and neonatal benefits.

### Children and Adolescents

The pediatric literature provides some of the earliest robust evidence for the obesity–iron link [6, 7]. Subsequent studies have shown that children and adolescents with overweight or obesity may have higher circulating hepcidin and lower iron status despite dietary iron intakes comparable with those of normal-weight peers, implicating inflammation and hepcidin-mediated iron restriction rather than intake alone [62, 63]. A more recent systematic review and meta-analysis of 42 pediatric studies reinforced this pattern, reporting a higher prevalence of iron deficiency in children with obesity (20.1% versus 16.1%) and greater odds of iron deficiency (odds ratio 1.64, 95% CI 1.22–2.21), together with lower hemoglobin, serum iron, and transferrin saturation but higher ferritin and hepcidin; the odds of iron-deficiency anemia were not significantly increased (odds ratio 0.78, 95% CI 0.43–1.43), consistent with functional, inflammation-driven iron restriction rather than absolute deficiency [64]. These findings support the consideration of iron status assessment in children and adolescents with obesity, particularly when symptoms, poor diet quality, rapid growth, heavy menstrual bleeding, or other risk factors are present. The same interpretive caveats that apply to adults also apply to younger populations: ferritin may be elevated by inflammation and should not be interpreted in isolation. This is important because iron deficiency in childhood has been associated with deficits in attention, memory, behavior, and cognitive development, some of which may persist even after correction [65]. Given the rising prevalence of pediatric obesity, pairing ferritin with an inflammation marker or using an inflammation-resistant index when available is particularly important when screening this population.

### Bariatric Surgery

Bariatric surgery introduces a distinct, anatomically driven threat to iron status, superimposed on the pre-existing inflammatory iron restriction of obesity. Reduced gastric acid can impair the reduction of ferric to ferrous iron, and procedures that bypass the duodenum and proximal jejunum remove the major sites of iron absorption [66]. Roux-en-Y gastric bypass and biliopancreatic diversion/duodenal switch generally carry higher risk of postoperative iron deficiency than purely restrictive procedures, but iron deficiency can also occur after sleeve gastrectomy because of reduced intake, lower gastric acid, intolerance of iron-rich foods, menstrual blood loss, and preexisting deficiency.

A systematic review reported that iron deficiency increased after Roux-en-Y gastric bypass, while the risk after sleeve gastrectomy was generally lower but not absent. Prevalence estimates varied widely according to procedure, follow-up duration, supplementation adherence, sex, menstrual status, and preoperative iron status [67]. Premenopausal women and patients with pre-existing iron deficiency are at the greatest risk. The American Society for Metabolic and Bariatric Surgery guidelines recommend routine postoperative micronutrient supplementation and surveillance. Lower-risk patients generally require at least 18 mg/day of iron from supplementation, whereas menstruating females and patients undergoing Roux-en-Y gastric bypass, sleeve gastrectomy, or biliopancreatic diversion/duodenal switch are commonly advised to receive 45–60 mg/day of elemental iron from all supplements combined; treatment of established deficiency requires higher doses and, in some cases, intravenous iron [68]. Here, too, ferritin must be interpreted in context. After surgery, patients may transition from an inflamed, hepcidin-mediated iron-restricted state toward a true absorptive deficiency as weight and inflammation decline. Therefore, monitoring should include ferritin, complete blood count, transferrin saturation, and clinical risk factors, with particular vigilance during the first postoperative year and continued long-term follow-up.

### Low- and Middle-Income Countries: the Double Burden

The overlap between obesity and iron deficiency is increasingly relevant in low- and middle-income countries undergoing the nutrition transition, where rising overweight and obesity coexist with endemic anemia and micronutrient deficiencies. This intraindividual “double burden of malnutrition,” defined as overweight or obesity and anemia or micronutrient deficiency in the same person, has been documented across multiple countries and appears to be especially important among women [69]. Emerging pediatric evidence also frames overnutrition as a potential risk factor for iron deficiency within this broader double-burden framework [65].

This double burden complicates public health iron programs. Blanket supplementation or fortification strategies designed for undernourished populations may be less effective when inflammation-driven functional deficiency predominates and may raise theoretical safety concerns in subgroups with hyperferritinemia, metabolic liver disease, or dysmetabolic iron loading. At the same time, iron deficiency remains highly prevalent and undertreatment carries serious maternal, developmental, and functional consequences. Therefore, population-level iron policy in transition settings increasingly needs to account for adiposity, inflammation, infection burden, dietary intake, and iron biomarkers, rather than relying on dietary intake or anemia prevalence alone. This issue is particularly relevant to global maternal and child nutrition needs.

## Management Implications

### Weight Loss and Inflammatory Iron Restriction

Because obesity-related iron restriction is mediated, in part, by low-grade inflammation and hepcidin elevation, weight loss may improve iron handling in some patients. In a small prospective, uncontrolled stable-isotope study, adults who underwent sleeve gastrectomy had significant reductions in body fat, IL-6, and serum hepcidin six months after surgery, accompanied by increased fractional dietary iron absorption [70]. This observation supports the biological pathway linking adiposity, inflammation, hepcidin, and impaired absorption, although the uncontrolled design means it should be interpreted as supportive rather than definitive proof of causality.

Consistent with this mechanism, a randomized controlled trial in young women with overweight or obesity and iron-deficiency anemia found that diet-induced weight loss improved iron status and related inflammatory and hepcidin levels [71]. Together, these studies suggest that lifestyle changes and surgical weight loss may improve iron bioavailability by reducing inflammatory hepcidin signaling. Therefore, weight loss interventions should be recognized not only as metabolic interventions but also as potential modifiers of obesity-associated iron dysregulation. However, their effect on iron status depends on the procedure type, dietary quality, baseline iron stores, menstrual blood loss, inflammation, and supplementation.

### Oral Versus Intravenous Iron

The biology of hepcidin in obesity has important therapeutic implications. As elevated hepcidin reduces ferroportin expression in enterocytes, oral iron absorption may be blunted in inflamed patients with obesity. In addition, high-dose or consecutive daily oral iron can transiently increase hepcidin levels and reduce fractional absorption from subsequent doses. Stable-isotope studies in iron-depleted and iron-deficient women suggest that single-dose alternate-day regimens can improve absorption efficiency compared to consecutive daily or divided dosing [72]. When oral iron is used, lower-dose single daily or alternate-day regimens may be reasonable, particularly when tolerability and adherence are concerns.

When inflammation and hepcidin levels are high, malabsorption is present, oral iron is not tolerated, or oral therapy has failed, intravenous iron bypasses the intestinal absorptive block and may provide more reliable repletion [8, 57]. However, intravenous iron should be used to treat confirmed or strongly suspected deficiency, not merely low transferrin saturation in a patient with adequate or excessive iron stores. The formulation choice is also important. Ferric carboxymaltose is associated with transient hypophosphatemia more often than some alternatives, including ferric derisomaltose, an issue that is particularly relevant in patients requiring repeated dosing [73]. Therefore, treatment decisions should be guided by the multi-marker assessment described above, including ferritin interpreted with an inflammation marker, transferrin saturation, and, where available, soluble transferrin receptor or reticulocyte hemoglobin. The response should be reassessed using hemoglobin and iron indices after repletion. The aim is to avoid both the undertreatment of true deficiency masked by inflammation-inflated ferritin and the overtreatment of patients whose low circulating iron reflects pure inflammatory sequestration without a need for additional iron.

### Iron Depletion in Dysmetabolic Iron Overload

At the iron-loaded pole, the temptation to remove iron should be resisted unless there is a clear indication of doing so. In a randomized controlled trial of 274 patients with dysmetabolic iron overload syndrome (DIOS), iron depletion by repeated phlebotomy to a low ferritin target did not improve glycemia, insulin resistance, or liver enzymes compared with lifestyle and dietary advice alone, and it was associated with greater fatigue [74]. Metabolic improvement occurred primarily in those who lost weight, irrespective of venesection.

Therefore, lifestyle modifications and management of metabolic risk should be considered first-line therapies for dysmetabolic hyperferritinemia and DIOS. Phlebotomy should not be used reflexively to treat obesity-associated hyperferritinemia. It may be considered only in selected patients with confirmed clinically meaningful iron overload after exclusion of hereditary hemochromatosis and other causes and with careful monitoring of symptoms, ferritin, transferrin saturation, hemoglobin, and hepatic status.

### Iron in the Era of Appetite-Suppressing Pharmacotherapy

The rapid adoption of glucagon-like peptide-1 (GLP-1) receptor agonists and dual incretin agonists has raised new questions regarding iron status. By reducing appetite, lowering total energy intake, and slowing gastric emptying, these therapies may reduce dietary iron intake or alter the conditions for absorption. Early dietary studies suggest that many users may fall short of the recommended protein and micronutrient intake during treatment [75, 76].

However, direct evidence that incretin therapy causes clinically meaningful iron deficiency remains limited, and its net effect is uncertain. Weight loss may reduce inflammation and hepcidin levels, which could improve iron bioavailability, whereas reduced intake or gastrointestinal intolerance could increase the deficiency risk in susceptible patients. Therefore, this issue should be framed as a research priority rather than as a basis for universal iron monitoring in all patients. Pending stronger data, attention to dietary adequacy is reasonable, particularly in menstruating women, pregnant patients, postbariatric patients, individuals with prior iron deficiency, those on restricted diets, those with gastrointestinal symptoms, and those with rapid weight loss.

## Conclusions and Future Directions

Several questions stand out for future work. The field still lacks a standardized, inflammation-robust biomarker panel for functional iron status; standardizing soluble transferrin receptor and hepcidin assays and validating reticulocyte hemoglobin and multi-marker algorithms in obesity would have immediate value. The model used to define iron deficiency also needs harmonizing, since our NHANES findings show it can materially alter the estimated burden.

Causal inference remains difficult because the relationship is bidirectional: adiposity restricts iron availability through inflammation and hepcidin, while iron can influence adipokines, oxidative stress, insulin sensitivity. Mendelian randomization suggests a small causal contribution of adiposity to iron-deficiency anemia but rests on assumptions; genetically informed designs could clarify directionality. The metabolic consequences of tissue iron loading need interventional testing, and iron handling during weight loss—lifestyle, pharmacological, or surgical—deserves systematic study. Special populations, including pregnant women, children and adolescents, and post-bariatric patients, need population-specific standards rather than extrapolation from healthy adult reference ranges.

Obesity and iron metabolism are linked through the hepcidin–ferroportin axis: adipose-derived inflammation, mainly via IL-6, raises hepcidin and restricts ferroportin-mediated iron export, reducing plasma and sequestering recycled iron. The same context can instead produce hyperferritinemia and dysmetabolic iron loading (notably in metabolic syndrome and MASLD. Iron may in turn shape adipocyte function and insulin sensitivity.

Clinically, obesity spans a continuum from functional iron deficiency to iron sufficiency, dysmetabolic hyperferritinemia or confirmed iron loading, but its defining problem is measurement: serum ferritin reflects inflammation as well as iron stores, so a normal or high value cannot be taken at face value. Reliable assessment requires multiple markers—ferritin with an inflammation marker plus transferrin saturation, soluble transferrin receptor, reticulocyte hemoglobin—while management targets reversible inflammation, matches iron repletion to absorption and hepcidin biology, and avoids reflexive depletion without confirmed overload.

## Key references

- Aguree S, Owora A, Hawkins M, Reddy MB. Iron Deficiency and Iron Deficiency Anemia in Women with and without Obesity: NHANES 2001-2006. Nutrients. 2023 May 11;15(10):2272. doi: 10.3390/nu15102272
  - ○ US national analysis showing higher odds of iron deficiency and iron-deficiency anemia in women with obesity and demonstrating that the choice of iron model materially changes prevalence estimates.
- Aguree S, Reddy MB. Inflammatory Markers and Hepcidin are Elevated but Serum Iron is Lower in Obese Women of Reproductive Age. Nutrients. 2021 Jan 14;13(1):217. doi: 10.3390/nu13010217.
  - ○ Direct characterization of the hypoferremia of obesity in reproductive-age women, linking elevated hepcidin and inflammation to lower circulating iron.
- Nemeth E, Rivera S, Gabayan V, Keller C, Taudorf S, Pedersen BK, Ganz T. IL-6 mediates hypoferremia of inflammation by inducing the synthesis of the iron regulatory hormone hepcidin. J Clin Invest. 2004 May;113(9):1271-6. doi: 10.1172/JCI20945.
  - ○ Establishes interleukin-6 as a sufficient driver of hepcidin and shows the hypoferremic response requires hepcidin, the mechanistic core of obesity-related iron restriction.
- Lynch S, Pfeiffer CM, Georgieff MK, Brittenham G, Fairweather-Tait S, Hurrell RF, McArdle HJ, Raiten DJ. Biomarkers of Nutrition for Development (BOND)-Iron Review. J Nutr. 2018 Jun 1;148(suppl_1):1001S-1067S. doi: 10.1093/jn/nxx036.
  - ○ Consensus framework for iron-biomarker selection that flags inflammation as the principal confounder of serum ferritin.
- Cepeda-Lopez AC, Osendarp SJ, Melse-Boonstra A, Aeberli I, Gonzalez-Salazar F, Feskens E, Villalpando S, Zimmermann MB. Sharply higher rates of iron deficiency in obese Mexican women and children are predicted by obesity-related inflammation rather than by differences in dietary iron intake. Am J Clin Nutr. 2011 May;93(5):975-83. doi: 10.3945/ajcn.110.005439.
  - ○ Shows that obesity-related inflammation, not lower dietary intake, accounts for higher iron deficiency in obesity.

## Data Availability

No datasets were generated or analysed during the current study.

## Declarations

This article does not contain any studies with human participants or animals performed by any of the authors.

### Use of Generative AI

The author(s) declare that no generative AI or AI-assisted technology was used in the writing of this manuscript.
